# Annual Performance Progression in Swimming Across Competition Levels and Race Distances

**DOI:** 10.3390/jfmk10030297

**Published:** 2025-07-31

**Authors:** Jesús J. Ruiz-Navarro, Dennis-Peter Born

**Affiliations:** 1 Aquatics Lab, Department of Physical Education and Sports, Faculty of Sport Sciences, University of Granada, 18011 Granada, Spain; jesusruiz@ugr.es; 2 Swiss Development Hub for Strength and Conditioning in Swimming, Swiss Swimming Federation, 3048 Worblaufen, Switzerland; 3 Department for Elite Sport, Swiss Federal Institute of Sport Magglingen, 2532 Magglingen, Switzerland; 4 Faculty of Science and Medicine, University of Fribourg, 1700 Fribourg, Switzerland

**Keywords:** athlete development, career trajectories, development program, competitive performance, long-term athlete development

## Abstract

**Objective:** As performance progression provides an essential indicator for talent selection and development, this study aimed to compare annual swimming performance progression between different competitive levels and to establish benchmarks for long-term athlete development. **Methods:** Annual best times of swimmers who competed up to the age of 21 years and achieved over 450 World Aquatics points were extracted from the database of European Aquatics. A total of 13,310 male and 7798 female pool swimmers of all race distances were grouped into three performance levels. **Results:** The results showed a continuous decline in annual performance progression throughout the years across all race distances (all *p* < 0.001) and in both sexes. There were differences between performance level across the age groups for all race distances in male swimmers, but only for the 100–400 m races in females (*p* < 0.05). Absolute performance showed significant main effects for level and age over all race distances for both sexes (all *p* < 0.001). **Conclusions:** Annual performance progression of swimmers consistently decreases across the competitive lifetime in both sexes, regardless of race distance and performance level. The event-specific benchmarks should be used as a framework to set realistic goals for both sexes and swimmers of different competitive levels, as well as to guide swimmers throughout their careers.

## 1. Introduction

In swimming, performance is quantified using the time a swimmer takes to cover a specific race distance. These race times are then used to determine qualification times and medal winners, as well as to select swimmers for regional, national, or international events. Benchmarks for average annual performance progressions support young swimmers and coaches in setting realistic goals based on age-specific qualification times. This is especially important in sports such as swimming, where goals are performance- rather than learning-oriented [1]; expectations that are set either too low or too high can lead to either insufficient training efforts or frustration due to repeated failures, respectively [2]. In comparison, realistic goals based on expected annual performance progression promote optimal athlete development.

Previous research used longitudinal designs to estimate performance progression and variability within and between seasons [3,4,5,6,7,8,9,10]. These data were also used to identify age thresholds that can, combined with race results, accurately predict adult age success (i.e., likelihood of swimmers winning medals or reaching finals at major international competitions [3,4,5,6,7,8,9,10]). These findings specifically describe swimmers’ developmental trajectories and shed light on the complex process of attaining peak performance in top elite swimmers. However, the majority of studies focused only on elite or high-performance-level swimmers [3,4,5,6,7,8,9,10], with a particular emphasis on athletes that participated in the Olympic Games [4]. Therefore, there is limited clarity regarding the career progression of swimmers of various performance levels, i.e., regional, national, and international swimmers.

Furthermore, previous studies have mainly focused on the performance development of single race distances—the 100 m being the most studied distance [8]. The common use of this distance is understandable, as the 100 m is considered the key swimming distance, as well as being represented across all four swimming strokes [11]. However, it is important to consider that aerobic and anaerobic contributions differ between the various distances and develop at different rates. These are affected by both the hormonal changes during puberty as well as training strategies throughout an athlete’s development [12]. Consequently, annual performance progression benchmarks for the more aerobic middle- and long-distance race distances cannot be derived from sprint events which rely on high anaerobic energy production [13,14]. Specific analyses are therefore required to assess performance progression across all race distances [5,7].

Due to the standardized competition conditions in swimming (e.g., water temperature, current, and pool length), compulsory electronic timekeeping, and the publicly accessible online databases, retrospective analysis of race results offers valuable insights into the development of competition performance [8]. These studies, using large data analyses, yield relevant information for development strategies, as they cover multiple generations of swimmers from different performance levels. In contrast to previous studies that focused on swimmers from specific nations [5,15], a global sample of swimmers from multiple nations would allow for the establishment of internationally representative benchmarks and norm values.

Therefore, the aim of this study was to examine and compare (1) the annual performance progression of swimmers from a representative sample of international swimmers and (2) absolute performance across different competitive levels and all freestyle race distances. Freestyle events were chosen to enable distance comparisons across the entire spectrum of race distances, i.e., 50 to 1500 m, of pool swimming competitions. The findings will contribute to both scientific understanding and practical application in swimming by expanding knowledge of performance development among competitive swimmers.

## 2. Materials and Methods

### 2.1. Subjects

A total of 169,788 races from 21,108 (13,310 males and 7798 females; age: ≥21 years old) individual freestyle pool swimmers were analyzed. Swimmers were categorized based on their highest World Aquatics points as follows: level 4 (450–650 points), level 3 (650–799 points), and level 2 (>800 points) [16]. Due to the small sample size of swimmers in the highest performance level (i.e., Level 1), Level 1 and 2 were pooled for this study. Moreover, as swimmers commonly compete in multiple events, the distribution of swimmers across the various performance levels and race distances is shown in Table 1. Race times were provided by the publicly accessible Swimrankings database (swimrankings.net), which includes swimmers from 165 countries. As only publicly available race times were used and analyzed anonymously, explicit written informed consent was not required. The study was preapproved by the institutional review board of the Swiss Federal Institute of Sport Magglingen (Reg.-Nr. 177_LSP_102022) and is in accordance with the World Medical Association’s Code of Conduct for medical research involving human subjects (Declaration of Helsinki).

### 2.2. Data Collection and Analysis

Race times between 2010 and 2023 were considered for this study. However, the 2020 database was excluded due to the impact of COVID-19. To ensure a high competition level, only results from long-course races (50 m pool length), which are the standard for major international championships, especially the Olympic Games, were analyzed. Furthermore, to focus on committed athletes who sustained their career into adulthood and consistently competed until peak performance age [17], swimmers were only included if they competed until the age of 21 years or older and achieved a performance level above 450 World Aquatics points [16].

Swimmers’ personal best times for each event were extracted and converted to World Aquatics points according to the official method of the governing body in swimming. The point system expresses race times relative to the prevailing world record (which equals 1000 points) and allows for the comparison of performances across various race distances [18]. To determine annual performance progression, swimmers’ annual best times were retrospectively extracted through adolescence from 9 to 21 years of age. To maximize the sample size, all swimmers from the database were included who had at least two years of recorded race times in any race distance. However, those two years had to be consecutive (e.g., if a swimmer was missing data at age 16, the progression from 15 to 17 was not considered). Annual performance progression (i.e., relative change in performance from one year to the next) was calculated using the following equation:

(1) $$Annual\;performance\;progression\left(\%\right)=\frac{swim\;time\;at\;age\;X-swim\;time\;at\;age\;(X+1)}{swim\;time\;at\;age\;(X+1)}\times 100$$

All data analyses were conducted in Python (version 3.11.5, Python Software Foundation, Beaverton, OR, USA) using the ‘pandas’ library for data analysis (version 2.0.3, pandas-dev/pandas, Zenodo, Genève, Switzerland).

### 2.3. Statistical Analysis

The data of male and female swimmers were analyzed separately and are presented as the mean ± standard deviation [19]. The Q-Q plot and Gaussian distribution of the histogram of the standardized residuals were used to confirm normality of the data. Logarithmic transformation was applied to data that were not normally distributed [20]. To prevent errors associated with negative numbers, a constant was added during the logarithmic transformation (i.e., all numbers were positive). Performance levels (2 vs. 3 vs. 4) across the ages (9 to 21) were compared by using a linear mixed model (LMM) analysis with fixed intercepts and restricted maximum likelihood (REML). Swimmers’ annual performance progression was used as the dependent variable, while performance levels and age were used as fixed factors with subject ID as a random factor. Bonferroni’s correction was used to correct post hoc tests for multiple pairwise comparisons. Additionally, in another step, the same model was applied with swimmers’ absolute race times as the dependent variable. Statistical procedures were carried out using Jamovi (The Jamovi Project 2022, version 2.3, retrieved from https://www.jamovi.org) and the level of significance was set at 0.05.

## 3. Results

**Relative performance** (annual performance progression) decreased continuously from 9 to 21 years of age over all events (main effect for all age categories *p* < 0.001), showing that the rate of improvement decreased as swimmers got older, independent of their performance level. However, the interaction effect between age and performance level indicates that level 2 swimmers maintained larger improvement rates over the years, while level 3 and particularly level 4 swimmers showed a continuous deterioration in swimming performance towards the oldest (20–21 year) age category, which is indicated by positive annual performance progression values (Table 2). This effect was primarily evident in male swimmers, while female swimmers showed similar performance progression across all performance levels (Table 3).

The comparison between race distances showed that performance in middle- and long-distance races plateaued (i.e., lack of improvement) at a younger age (in the 17–18-year age category) compared to sprint races (19–20 year). Moreover, female swimmers’ performance plateaued earlier than that of male swimmers in 100, 200, and 400 m races (*p* ≤ 0.012), with similar trends for the other race distances as indicated by the interaction effects (*p* = 0.136–0.55).

**Figure 1:**
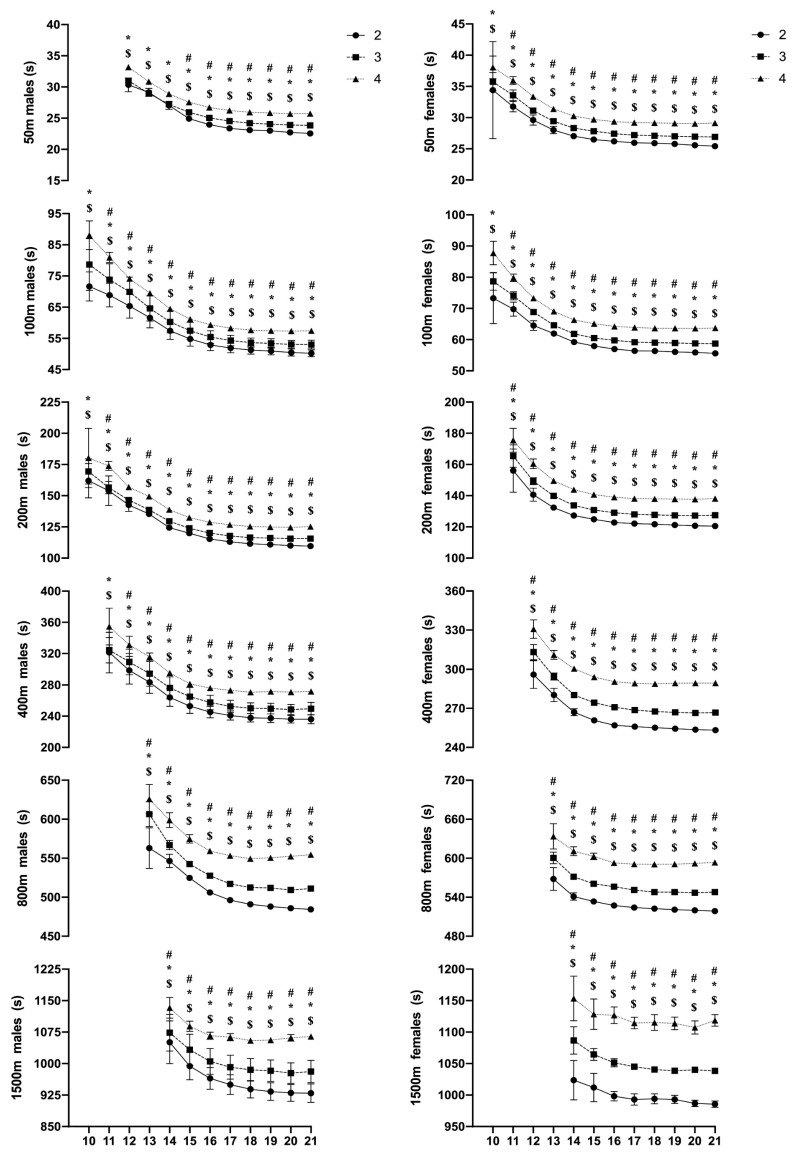
The absolute performance development of male and female swimmers across the various age categories and performance levels. # A significant difference between performance level 2 and level 3 swimmers. * A significant difference between performance level 2 and level 4 swimmers. $ A significant difference between performance level 3 and level 4 swimmers.

**Absolute performance** (race times) showed significant main effects for performance level and age across all race distances and for both sexes (all *p* < 0.001). Specifically, male level 2 swimmers exhibited faster race times than level 3 swimmers across all age groups (except in the youngest age groups (10–12 years (*p* > 0.05))) and events (*p* < 0.001), which is indicated by interaction effect (performance level × age). Additionally, male level 2 swimmers were faster than level 4 swimmers across all ages and events (*p* < 0.001). Similarly, female level 2 swimmers showed faster race times compared to level 3 swimmers, except during the 10-year age category across the 50 and 100 m events (*p* > 0.05) (Figure 1).

## 4. Discussion

The aim of the present study was to examine and compare the annual performance progression of male and female swimmers across various competitive levels (performance level 4 (450–650 points), level 3 (650–799 points), level 2 (>800 points)) over all freestyle race distances. The main findings are that annual performance progression (yearly improvement) continuously decreases across the swimmers’ competitive lifetime in both sexes and across all race distances. High-performance swimmers (i.e., level 2) achieve faster race times (better absolute performance) than level 3 and level 4 swimmers from the beginning of their competitive career. Additionally, level 2 swimmers keep developing towards the older age categories and exhibit greater annual performance progression even during older age categories. In contrast, level 3 and especially level 4 swimmers show an early plateau and even experience a progressive decline in performance as they approach the oldest age categories.

There is a general decline in annual performance progression from the beginning of the swimmers’ competitive careers towards peak performance age. This effect is evident regardless of sex, performance level, and race distance (Table 2 and Table 3) and aligns with previous findings reported in top Portuguese male swimmers [5]. Interestingly, this annual performance progression declines despite the increase in the number of swimming sessions per week, hours of training per day, and the inclusion of additional dry-land training sessions [21,22]. During the initial stages of a swimmers’ career, notable improvements in motor learning skills during children’s formative years and biological maturation [23,24,25] result in the initially steep performance improvements. These enhancements can be explained by the improvement in swimming technique and biomechanical key performance indicators [26]. During older age categories, performance enhancements occur as a result of physiological and neuromuscular adaptations as well as the refinement of biomechanical abilities [26]. As swimmers may be discouraged by their decline in progression, coaches should clearly communicate the expected performance improvements, particularly towards later junior age. The present study quantifies average annual performance progression for each age category and helps set realistic goals and expectations, which is particularly important when approaching peak performance age.

Annual progression follows a similar pattern in swimmers of all three performance levels. Trend analysis showed slightly greater, but not significant, improvements in level 3 and 4 swimmers during the first years of their competitive career (9–13 years of age). However, a reversal point is evident around the age of 14 years, where level 2 swimmers tend to show higher, but also non-significant, annual progression in all events. More importantly, both male and female level 2 swimmers’ performances continued to improve up to 21 years of age, while level 3 and 4 swimmers’ performances declined during the last two years (20–21-year age category) before peak performance age. The same patterns were observed across all race distances, despite the slightly smaller performance improvements in long-distance events. Therefore, the biomechanical and physiological demands of short-, middle-, and long-distance races may not impact annual performance progression [5]. The high-volume training regimes that aim to develop aerobic capacity are particularly common during junior age [27] and initially result in a higher performance level (higher World Aquatics points) in long- compared to middle- and short-distance races of top elite swimmers [10]. However, as this energetic system reaches its maximal capacity earlier than the anaerobic system, it may partly account for the slightly reduced performance improvements observed in long-distance swimmers nearing their peak performance age.

Despite the slower improvement rates in level 2 swimmers at the beginning of their swimming career, their absolute performance was better than that of level 3 swimmers. Moreover, both level 2 and 3 swimmers outperformed level 4 swimmers across short, middle, and long distances, and this trend was seen in both sexes irrelevant of age category. As previously shown, this performance gap even increases from the ages of 14–15 in males and 12–13 in females [8]. During those age categories, the present study shows a maximal annual performance improvement of 4–7%, which underlines the necessity of a sufficiently high performance level during junior age [8], so to swimmers can stand a chance as a high-performance swimmer when approaching their early 20s [9]. According to the present findings, higher-ranked swimmers have to have better absolute performance at an early age and must distinguish themselves from their lower-ranked peers after the most critical stage (i.e., puberty) of their biological development. As differences in motor skills, i.e., better and more efficient swimming technique [28,29,30], largely contribute to the differences in performance levels, these findings highlight the importance of technical skill acquisition during the early years of a swimmer’s career. Emphasizing physiological development with high training volumes and intensities at an early age may promote quick success. However, the technical deficiencies due to insufficient development of swimming technique may result in an earlier cessation of the progression rate, as the potential of further physiological development is exhausted.

Due to the high reliance on technical key performance indicators, swimming may require earlier specialization compared to other sports in order to accumulate the necessary years of training and competition participation [31,32]. However, the findings of the present study should be used as benchmarks for the functional development of swimming performance towards peak performance age. Coaches and federation officials should use annual performance progression to guide their swimmers throughout their career, placing adequate attention on their technical education, rather than fostering physiological development too early in their career. Also, the decline in annual performance progression should be acknowledged in a functional long-term development plan [32], which should still enable continuous progression towards peak performance age without resulting in demotivation and, in the worst case, withdrawal from sporting activities [33]. This is particularly relevant for female swimmers, who are more likely to reach high performance levels at an earlier age than their male counterparts and hence go through the experiences of stagnation and performance plateaus at a younger age.

A strength of the present study is the large dataset that allows for the analysis of the global swimming population rather than using a sub-sample. However, retrospective analyses of race times, such as in the present study, do not distinguish between infrastructural and socio-economic factors that may differ between countries, nor do they consider differences in coaches’ qualifications, biological growth, or training regimes (i.e., number of training sessions per week, hours per session, periodization, and competition frequency). As all of these factors influence the study outcomes, the big data approach aims to compensate for these variabilities. Coaches are advised to use not only the present mean values but also the corresponding 95% confidence interval to enhance development corridors throughout the careers of young swimmers towards peak performance age, enabling them to account for individual variation across long-term athlete development. Future studies should explore the evolution of swimmers while considering the abovementioned influential factors.

## 5. Conclusions

In conclusion, regardless of performance level, the annual performance progression of swimmers consistently decreases across the competitive lifetime of both sexes across all distances. Major differences in annual performance progression were found during early junior age and close to peak performance age (i.e., 20–21 years) across the three performance levels. As such, during early junior age, level 2 swimmers showed a slower rate of annual performance progression. However, shortly before peak performance age (20–21-year age category), level 2 swimmers still progressed, while the performance of level 3 and 4 swimmers plateaued or even declined. These benchmarks may serve as a valuable tool for setting realistic goals within long-term athlete development frameworks. Nevertheless, due to individual differences among swimmers—shaped by various influencing factors—coaches are advised to interpret and apply these benchmarks with consideration of each athlete’s unique trajectory and variability.

## Figures and Tables

**Table 1:** Distribution of swimmers based on their competitive level and event distances.

Performance Level	Level 4		Level 3		Level 2	
**Distance**	**M**	**F**	**M**	**F**	**M**	**F**
50 m	5849	3196	2645	1707	264	214
100 m	4452	2661	3925	2151	824	363
200 m	2601	1457	2889	1773	515	395
400 m	1108	920	1759	1211	760	255
800 m	498	384	862	687	318	194
1500 m	303	150	691	287	356	86

M: males; F: females.

**Table 2:** Male swimmers’ annual performance progression (mean ± standard deviation [95% confidence interval (gray)]) across the age categories [years] at performance level 2 (≥800 points), level 3 (650–799 points), and level 4 (450–649 points). Swimmers were ranked based on their personal best at peak performance age in each race distance.

Performance Level		Age Categories [Years]												Linear Mixed Model Analysis		
		9–10	10–11	11–12	12–13	13–14	14–15	15–16	16–17	17–18	18–19	19–20	20–21			
**50 m** **n = 8751**	**2**	-	-	−7.6 ± 2.96 [−10.71, −4.5]	−5.14 ± 4.08 [−7.5, −2.78]	−5.68 ± 2.35 [−6.63, −4.73]	−5.83 ± 3.34 [−6.74, −4.91]	−3.70 ± 2.17 # [−4.16, −3.24]	−2.93 ± 2.08 [−3.31, −2.55]	−1.94 ± 2.28 [−2.33, −1.54]	−0.73 ± 1.81 # [−1.02, −0.43]	−1.28 ± 1.88 [−1.57, −1]	−0.92 ± 1.66 [−1.16, −0.69]	R^2^_c_= 0.36 ICC = 1.08^−15^	(a) F_[9|28282] = 342.4_	*p* < 0.001
	**3**	-	-	−7.34 ± 7.13 [−8.74, −5.94]	−7.02 ± 4.65 [−7.7, −6.35]	−6.42 ± 3.6 [−6.82, −6.03]	−4.72 ± 2.79 # [−4.94, −4.49]	−3.46 ± 2.39 # [−3.62, −3.3]	−2.20 ± 2.29 # [−2.33, −2.07]	−1.66 ± 2.16 # [−1.77, −1.55]	−0.78 ± 2.08 # [−0.89, −0.67]	−0.72 ± 2.06 [−0.82, −0.61]	−0.26 ± 2.03 # [−0.35, −0.16]		(b) F_[2|28282] = 0.5_	*p* = 0.600
	**4**	-	-	−8.20 ± 5.11 [−8.82, −7.58]	−7.19 ± 4.24 # [−7.57, −6.81]	−6.41 ± 3.68 # [−6.65, −6.16]	−4.61 ± 3.02 # [−4.76, −4.45]	−3.29 ± 2.76 # [−3.41, −3.17]	−2.11 ± 2.39 # [−2.2, −2.02]	−1.34 ± 2.33 # [−1.43, −1.25]	−0.63 ± 2.28 # [−0.72, −0.53]	−0.51 ± 2.28 [−0.6, −0.42]	−0.05 ± 2.55 # * [−0.14, 0.04]		(c) F_[18|28282] = 2.9_	*p* < 0.001
**100 m** **n = 9201**	**2**	−12.01 ± 2.11 [−13.96, −10.1]	−7.42 ± 5.25 # [−10.21, −4.62]	−7.54 ± 4.38 [−8.85, −6.22]	−7.02 ± 3.25 [−7.77, −6.28]	−6.57 ± 3.35 [−7.2, −5.95]	−4.86 ± 2.48 # [−5.2, −4.52]	−3.50 ± 2.25 # [−3.75, −3.24]	−2.24 ± 1.97 # [−2.43, −2.05]	−1.85 ± 2.13 [−2.04, −1.66]	−0.86 ± 1.82 # [−1.02, −0.7]	−0.9 ± 1.74 [−1.05, −0.75]	−0.60 ± 1.73 [−0.74, −0.46]	R^2^_c_= 0.43 ICC = 1.64^−14^	(a) F_[11|32349] = 1153_	*p* < 0.001
	**3**	−12.99 ± 5.93 [−15.62, −10.4]	−9.54 ± 5.65 [−10.77, −8.31]	−8.8 ± 5.35 [−9.57, −8.03]	−7.49 ± 4.25 # [−7.93, −7.06]	−6.74 ± 3.47 # [−7.01, −6.47]	−4.80 ± 2.90 # [−4.97, −4.62]	−3.50 ± 2.48 # [−3.63, −3.38]	−2.10 ± 2.11 # [−2.2, −2.01]	−1.54 ± 2.11 # [−1.63, −1.45]	−0.62 ± 2.21 # [−0.72, −0.53]	−0.69 ± 2.07 [−0.78, −0.6]	−0.08 ± 2.08 * [−0.16, 0]		(b) F_[2|32349] = 7.93_	*p* < 0.001
	**4**	−12.00 ± 5.00 [−14.34, −9.66]	−10.28 ± 5.12 * [−11.36, −9.2]	−8.61 ± 5.88 # [−9.39, −7.83]	−8.00 ± 4.48 [−8.4, −7.6]	−7.10 ± 3.96 # [−7.37, −6.83]	−5.15 ± 3.32 # [−5.33, −4.97]	−3.59 ± 3.13 # [−3.74, −3.45]	−2.18 ± 2.65 # [−2.29, −2.06]	−1.38 ± 2.54 # [−1.49, −1.27]	−0.60 ± 2.34 # [−0.71, −0.49]	−0.44 ± 2.46 [−0.56, −0.32]	0.02 ± 2.61 * [−0.09, 0.13]		(c) F_[22|32349] = 5.37_	*p* < 0.001
**200 m** **n = 6005**	**2**	−6.25 ± 3.90 [−12.45, −0.04]	−9.15 ± 5.73 [−13.93, −4.36]	−6.48 ± 4.13 [−8.54, −4.43]	−6.21 ± 3.38 [−7.34, −5.08]	−6.89 ± 3.37 [−7.71, −6.06]	−4.59 ± 2.50 # [−5.01, −4.17]	−3.52 ± 2.59 [−3.86, −3.17]	−2.26 ± 2.13 # [−2.51, −2.02]	−1.52 ± 2.24 [−1.76, −1.27]	−0.81 ± 2.10 [−1.03, −0.58]	−0.72 ± 2.25 [−0.95, −0.48]	−0.39 ± 2.05 [−0.6, −0.18]	R^2^_c_= 0.37 ICC = 9.37^−15^	(a) F_[11|20519] = 623.68_	*p* < 0.001
	**3**	−10.24 ± 7.21 [−16.26, −4.21]	−8.44 ± 6.39 [−10.91, −5.96]	−8.83 ± 5.50 * [−10.07, −7.59]	−7.28 ± 4.23 [−7.91, −6.65]	−6.63 ± 3.31 [−6.97, −6.28]	−4.77 ± 2.86 # [−4.98, −4.57]	−3.35 ± 2.68 # [−3.51, −3.2]	−1.99 ± 2.14 # [−2.1, −1.88]	−1.34 ± 2.05 # [−1.44, −1.24]	−0.44 ± 2.22 # [−0.55, −0.33]	−0.47 ± 2.11 [−0.57, −0.37]	0.12 ± 2.55 # [0.01, 0.24]		(b) F_[2|20519] = 6.5_	*p* = 0.002
	**4**	−11.27 ± 5.31 [−19.72, −2.81]	−8.25 ± 3.98 [−9.82, −6.67]	−8.87 ± 3.78 [−9.79, −7.96]	−7.13 ± 4.32 # [−7.8, −6.45]	−7.29 ± 3.87 [−7.68, −6.9]	−4.95 ± 3.56 # [−5.22, −4.69]	−3.38 ± 3.04 # [−3.58, −3.19]	−1.99 ± 2.89 # [−2.16, −1.82]	−1.21 ± 2.91 # [−1.38, −1.04]	−0.35 ± 2.86 # [−0.53, −0.17]	−0.44 ± 2.73 [−0.61, −0.26]	0.19 ± 3.36 # [0, 0.37]		(c) F_[22|20519] = 3.4_	*p* < 0.001
**400 m** **n = 3627**	**2**	-	−8.37 ± 2.46 [−10.43, −6.32]	−6.01 ± 3.39 [−7.51, −4.5]	−6.48 ± 3.65 [−7.48, −5.47]	−5.88 ± 3.06 [−6.43, −5.33]	−4.48 ± 2.51 # [−4.82, −4.14]	−3.23 ± 2.21 # [−3.48, −2.99]	−1.97 ± 1.96 # [−2.15, −1.78]	−1.44 ± 1.75 [−1.6, −1.28]	−0.41 ± 1.92 # [−0.58, −0.24]	−0.62 ± 2.05 [−0.8, −0.44]	−0.11 ± 2.3 [−0.3, 0.08]	R^2^_c_= 0.33 ICC = 2.64^−15^	(a) F_[8|12638] = 235.6_	*p* < 0.001
	**3**	-	−9.43 ± 5.00 [−12.78, −6.07]	−9.05 ± 3.41 * [−10.28, −7.82]	−6.94 ± 3.65 # [−7.59, −6.29]	−6.45 ± 3.44 [−6.86, −6.03]	−4.55 ± 3.01 # [−4.81, −4.28]	−3.07 ± 2.68 # [−3.27, −2.87]	−1.94 ± 2.46 # [−2.1, −1.78]	−1.12 ± 2.25 # [−1.26, −0.97]	−0.37 ± 2.36 # [−0.52, −0.21]	−0.28 ± 2.41 [−0.44, −0.12]	0.33 ± 2.74 # * [0.17, 0.49]		(b) F_[2|12638] = 3.2_	*p* = 0.968
	**4**	-	−6.53 ± 4.60 [−11.36, −1.69]	−8.94 ± 4.19 * [−10.6, −7.29]	−7.17 ± 4.22 [−8.09, −6.25]	−6.40 ± 3.76 [−6.97, −5.84]	−4.47 ± 3.45 # [−4.88, −4.07]	−2.72 ± 3.82 # [−3.1, −2.34]	−1.80 ± 3.83 # [−2.15, −1.46]	−1.20 ± 2.86 [−1.46, −0.94]	−0.07 ± 2.90 # [−0.36, 0.23]	−0.23 ± 3.12 [−0.55, 0.1]	0.17 ± 3.51 # * [−0.13, 0.46]		(c) F_[16|12638] = 1.8_	*p* = 0.034
**800 m** **n = 1678**	**2**	-	-	-	−8.31 ± 4.72 [−14.17, −2.44]	−4.73 ± 2.42 [−5.68, −3.77]	−4.31 ± 2.52 [−4.89, −3.73]	−3.44 ± 2.15 [−3.83, −3.05]	−2.41 ± 2.01 [−2.72, −2.11]	−1.3 ± 2.15 [−1.61, −0.99]	−0.7 ± 2.08 [−1.0, −0.4]	−0.63 ± 2.01 [−0.91, −0.35]	−0.41 ± 2.12 [−0.68, −0.13]	R^2^_c_= 0.30 ICC = 0.01	(a) F_[9|5426] = 201.3_	*p* < 0.001
	**3**	-	-	-	−4.62 ± 3.47 [−6.29, −2.95]	−6.79 ± 3.16 [−7.6, −5.98]	−4.49 ± 2.96 [−4.93, −4.05]	−3.03 ± 2.8 # [−3.35, −2.71]	−2.12 ± 2.42 [−2.36, −1.89]	−1.06 ± 2.18 [−1.26, −0.86]	−0.29 ± 2.31 [−0.5, −0.07]	−0.36 ± 2.26 [−0.57, −0.15]	0.21 ± 2.35 [0.02, 0.41]		(b) F_[2|3673] = 0.02_	*p* = 0.982
	**4**	-	-	-	−6.4 ± 3.18 [−8.16, −4.64]	−6.67 ± 3.37 [−7.67, −5.67]	−4.35 ± 2.59 [−4.82, −3.87]	−2.95 ± 2.95 # [−3.42, −2.47]	−1.86 ± 3.0 [−2.27, −1.44]	−1.04 ± 2.44 [−1.36, −0.72]	−0.28 ± 2.61 [−0.65, 0.09]	−0.11 ± 3.3 [−0.62, 0.4]	0.40 ± 3.57 [−0.03, 0.84]		(c) F_[18|5311] = 2.13_	*p* = 0.003
**1500 m** **n = 1350**	**2**	-	-	-	-	−4.78 ± 2.68 [−5.97, −3.59]	−4.45 ± 2.62 [−5.02, −3.87]	−3.07 ± 1.92 # [−3.4, −2.74]	−1.95 ± 1.8 # [−2.21, −1.69]	−1.27 ± 1.89 [−1.53, −1.01]	−0.74 ± 1.83 [−0.99, −0.49]	−0.47 ± 1.7 [−0.68, −0.25]	−0.09 ± 1.87 [−0.31, 0.14]	R^2^_c_= 0.30 ICC = 0.02	(a) F_[7|4197] = 204.7_	*p* < 0.001
	**3**	-	-	-	-	−6.04 ± 2.75 [−6.85, −5.23]	−4.23 ± 2.73 # [−4.69, −3.76]	−2.83 ± 2.67 # [−3.17, −2.5]	−1.61 ± 2.3 # [−1.86, −1.36]	−0.99 ± 2.19 [−1.21, −0.76]	−0.3 ± 2.08 # [−0.52, −0.08]	−0.45 ± 2.04 [−0.66, −0.23]	0.15 ± 2.02 [−0.04, 0.34]		(b) F_[2|1569] = 1.1_	*p* = 0.339
	**4**	-	-	-	-	−7.1 ± 4.13 * [−9.08, −5.11]	−4.48 ± 3.52 # [−5.4, −3.57]	−2.61 ± 2.76 # [−3.19, −2.02]	−1.49 ± 3.24 [−2.1, −0.89]	−0.77 ± 2.52 [−1.22, −0.33]	−0.54 ± 2.34 [−0.97, −0.1]	−0.15 ± 2.72 [−0.7, 0.39]	0.17 ± 2.69 [−0.26, 0.6]		(c) F_[14|4305] = 1.9_	*p* = 0.021

Linear mixed model analysis. (a) Main effect: age category. (b) Main effect: performance level. (c) Interaction effect: age category x performance level. Bonferroni post hoc comparison: # significant difference compared to previous age category. * significant difference compared to performance level 2.

**Table 3:** Female swimmers’ annual performance progression (mean ± standard deviation [95% confidence interval (gray)]) across the age categories [years] at performance level 2 (≥800 points), level 3 (650–799 points), and level 4 (450–649 points). Swimmers were ranked based on their personal best at peak performance age in each race distance.

Performance Level		Age Categories [Years]												Linear Mixed Model Analysis		
		9–10	10–11	11–12	12–13	13–14	14–15	15–16	16–17	17–18	18–19	19–20	20–21			
**50 m** **n = 5117**	**2**	−15.02 ± 5.17 [−27.87, −2.17]	−8.53 ± 4.45 # [−11.72, −5.34]	−6.69 ± 3.84 [−8.82, −4.57]	−6.10 ± 2.54 [−7.36, −4.83]	−3.37 ± 2.60 # [−4.21, −2.53]	−2.37 ± 2.16 [−2.9, −1.83]	−1.76 ± 2.07 [−2.17, −1.35]	−1.30 ± 1.96 [−1.64, −0.95]	−0.65 ± 1.82 [−0.98, −0.33]	−0.70 ± 2.01 [−1.06, −0.34]	−0.86 ± 1.68 [−1.13, −0.58]	−0.65 ± 1.61 [−0.9, −0.41]	R^2^_c_= 0.34 ICC = 1.44^−14^	(a) F_[11|18231] = 312.2_	*p* < 0.001
	**3**	−12.19 ± 5.08 [−15.27, −9.12]	−8.51 ± 4.95 # [−9.98, −7.04]	−7.24 ± 3.77 [−8.1, −6.37]	−5.77 ± 4.19 # [−6.37, −5.17]	−3.68 ± 2.63 # [−3.95, −3.41]	−2.33 ± 2.26 # [−2.52, −2.15]	−1.41 ± 1.95 # [−1.55, −1.27]	−0.91 ± 2.08 [−1.05, −0.78]	−0.49 ± 1.99 [−0.62, −0.36]	−0.31 ± 2.10 [−0.46, −0.17]	−0.31 ± 2.00 [−0.44, −0.17]	−0.21 ± 2.04 [−0.33, −0.09]		(b) F_[2|18231] = 3.3_	*p* = 0.037
	**4**	−12.12 ± 6.08 [−15.14, −9.1]	−9.03 ± 4.14 # * [−9.91, −8.16]	−7.26 ± 3.98 # [−7.9, −6.63]	−5.86 ± 3.35 # [−6.2, −5.51]	−3.74 ± 2.99 # [−3.96, −3.51]	−2.33 ± 2.75 # [−2.5, −2.17]	−1.24 ± 2.37 # [−1.37, −1.12]	−0.69 ± 2.40 # [−0.81, −0.57]	−0.25 ± 2.30 # [−0.37, −0.14]	−0.09 ± 2.28 [−0.22, 0.04]	−0.12 ± 2.30 [−0.25, 0.02]	0.21 ± 2.45 [0.09, 0.33]		(c) F_[22|18231] = 1.3_	*p* = 0.136
**100 m** **n = 5175**	**2**	−20.81 ± 16.55 [−169.5, 127.92]	−8.34 ± 4.11 # [−10.39, −6.3]	−7.53 ± 3.33 [−8.88, −6.19]	−5.95 ± 3.68 [−6.99, −4.92]	−3.90 ± 2.76 # [−4.47, −3.34]	−2.50 ± 1.85 # [−2.81, −2.19]	−1.79 ± 1.86 [−2.06, −1.51]	−1.15 ± 1.84 [−1.4, −0.9]	−0.46 ± 1.77 [−0.7, −0.23]	−0.74 ± 2.03 [−1.01, −0.47]	−0.43 ± 1.78 [−0.66, −0.2]	−0.44 ± 1.95 [−0.67, −0.2]	R^2^_c_= 0.41 ICC = 3.33^−15^	(a) F_[11|19776] = 593.9_	*p* < 0.001
	**3**	−14.35 ± 6.94 * [−17.8, −10.9]	−9.81 ± 4.93 # [−10.91, −8.72]	−8.74 ± 4.56 [−9.48, −8]	−6.13 ± 3.70 # [−6.55, −5.71]	−4.44 ± 3.08 # [−4.71, −4.17]	−2.42 ± 2.46 # [−2.6, −2.25]	−1.57 ± 2.00 # [−1.7, −1.45]	−1.08 ± 2.08 [−1.2, −0.96]	−0.44 ± 1.94 # [−0.55, −0.33]	−0.06 ± 2.29 [−0.2, 0.07]	−0.23 ± 2.01 [−0.35, −0.11]	−0.06 ± 2.33 [−0.18, 0.06]		(b) F_[2|19776] = 22.6_	*p* < 0.001
	**4**	−13.29 ± 6.22 * [−16.05, −10.53]	−10.46 ± 5.24 # [−11.53, −9.4]	−8.95 ± 4.94 # [−9.66, −8.25]	−6.40 ± 3.95 # [−6.78, −6.03]	−4.33 ± 3.39 # [−4.59, −4.08]	−2.48 ± 2.97 # [−2.66, −2.29]	−1.46 ± 2.69 # [−1.61, −1.31]	−0.76 ± 2.68 # [−0.9, −0.62]	−0.32 ± 2.70 [−0.47, −0.17]	0.10 ± 2.64 [−0.07, 0.27]	−0.16 ± 2.69 [−0.33, 0.02]	0.20 ± 2.78 [0.05, 0.35]		(c) F_[22|19776] = 6.9_	*p* < 0.001
**200 m** **n = 3625**	**2**	-	−8.86 ± 4.31 [−12.85, −4.87]	−7.01 ± 3.80 [−8.97, −5.06]	−5.58 ± 2.79 [−6.49, −4.66]	−4.16 ± 2.91 [−4.81, −3.5]	−2.37 ± 2.03 # [−2.71, −2.03]	−1.84 ± 1.93 [−2.12, −1.56]	−0.88 ± 1.76 [−1.11, −0.65]	−0.59 ± 1.81 [−0.82, −0.36]	−0.42 ± 1.97 [−0.67, −0.16]	−0.44 ± 1.71 [−0.65, −0.22]	−0.14 ± 1.87 [−0.36, 0.07]	R^2^_c_= 0.33 ICC = 0.00	(a) F_[10|13352] = 425.99_	*p* < 0.001
	**3**	-	−11.56 ± 5.61 [−13.83, −9.3]	−8.14 ± 3.59 # [−8.96, −7.32]	−6.52 ± 3.72 # [−7.04, −5.99]	−4.32 ± 3.11 # [−4.64, −4.01]	−2.51 ± 2.35 # [−2.7, −2.32]	−1.53 ± 1.96 # [−1.67, −1.39]	−0.90 ± 2.00 # [−1.02, −0.77]	−0.36 ± 1.97 # [−0.48, −0.24]	−0.17 ± 2.21 [−0.31, −0.02]	−0.05 ± 2.27 [−0.2, 0.09]	0.10 ± 2.32 [−0.04, 0.23]		(b) F_[2|13352] = 12.35_	*p* < 0.001
	**4**	-	−8.48 ± 7.64 [−11.71, −5.25]	−7.89 ± 4.60 [−9.09, −6.68]	−6.57 ± 3.62 # [−7.14, −6]	−4.15 ± 3.46 # [−4.52, −3.78]	−2.43 ± 2.78 # [−2.67, −2.19]	−1.57 ± 2.65 # [−1.78, −1.36]	−0.91 ± 2.64 # [−1.11, −0.72]	−0.31 ± 2.75 # [−0.52, −0.1]	−0.08 ± 2.97 [−0.33, 0.18]	−0.08 ± 2.59 [−0.31, 0.15]	0.38 ± 2.82 [0.17, 0.59]		(c) F_[22|13354] = 2.9_	*p* < 0.001
**400 m** **n = 2386**	**2**	-	-	−6.93 ± 1.93 [−8.54, −5.32]	−5.26 ± 3.13 [−6.49, −4.02]	−3.89 ± 2.24 [−4.5, −3.29]	−2.63 ± 2.19 [−3.1, −2.15]	−1.82 ± 1.75 [−2.14, −1.49]	−0.89 ± 1.73 [−1.18, −0.6]	−0.48 ± 1.52 [−0.71, −0.24]	−0.38 ± 1.69 [−0.65, −0.12]	−0.21 ± 1.72 [−0.48, 0.05]	−0.07 ± 2.00 [−0.36, 0.22]	R^2^_c_= 0.29 ICC = 0.00	(a) F_[10|8712] = 229.2_	*p* < 0.001
	**3**	-	-	−6.93 ± 4.01 [−8.1, −5.75]	−6.00 ± 3.66 [−6.61, −5.39]	−3.99 ± 2.97 # [−4.36, −3.63]	−2.40 ± 2.62 # [−2.66, −2.13]	−1.43 ± 1.98 # [−1.6, −1.25]	−0.83 ± 2.33 [−1.02, −0.65]	−0.42 ± 2.20 [−0.6, −0.25]	−0.21 ± 2.31 [−0.39, −0.02]	−0.14 ± 2.22 [−0.32, 0.04]	0.09 ± 2.25 [−0.06, 0.24]		(b) F_[2|8712] = 5.2_	*p* = 0.006
	**4**	-	-	−8.72 ± 5.14 [−10.34, −7.1]	−6.31 ± 3.99 # [−7.07, −5.55]	−4.07 ± 3.54 # [−4.52, −3.63]	−2.42 ± 3.12 # [−2.75, −2.09]	−1.20 ± 2.83 # [−1.47, −0.92]	−0.84 ± 2.78 [−1.11, −0.58]	−0.34 ± 2.88 [−0.62, −0.05]	−0.23 ± 2.74 [−0.53, 0.06]	0.18 ± 2.85 [−0.14, 0.49]	0.25 ± 3.06 [−0.04, 0.53]		(c) F_[20|8712] = 1.9_	*p* = 0.012
**800 m** **n = 1265**	**2**	-	-	-	−3.94 ± 3.06 [−6.13, −1.75]	−3.93 ± 2.01 [−4.6, −3.26]	−1.99 ± 1.76 # [−2.44, −1.54]	−1.53 ± 1.86 [−1.95, −1.11]	−0.96 ± 1.73 [−1.3, −0.62]	−0.51 ± 1.45 [−0.78, −0.24]	−0.33 ± 1.45 [−0.61, −0.06]	−0.36 ± 1.56 [−0.65, −0.08]	−0.23 ± 1.75 [−0.52, 0.06]	R^2^_c_= 0.22 ICC = 0.00	(a) F_[9|4284] = 108.9_	*p* < 0.001
	**3**	-	-	-	−6.06 ± 3.15	−3.91 ± 2.74 #	−2.44 ± 2.47 #	−1.21 ± 2.04 #	−0.83 ± 1.87	−0.52 ± 1.99	−0.18 ± 1.96	−0.18 ± 1.95	0.04 ± 2.31		(b) F_[2|4284] = 0.105_	*p* = 0.902
					[−7.13, −4.99]	[−4.43, −3.39]	[−2.78, −2.09]	[−1.45, −0.97]	[−1.03, −0.63]	[−0.73, −0.31]	[−0.39, 0.03]	[−0.39, 0.03]	[−0.17, 0.26]			
	**4**	-	-	-	−5.44 ± 2.97 [−7.02, −3.86]	−3.59 ± 3.17 [−4.41, −2.77]	−2.39 ± 3.09 [−2.99, −1.8]	−1.31 ± 2.56 # [−1.73, −0.89]	−0.63 ± 2.59 [−1.04, −0.23]	−0.64 ± 2.32 [−1.01, −0.27]	−0.60 ± 2.42 [−1.01, −0.18]	0.22 ± 2.42 [−0.19, 0.63]	0.29 ± 2.67 [−0.08, 0.66]		(c) F_[18|4284] = 1.6_	*p* = 0.055
**1500 m** **n =523**	**2**	-	-	-	-	−3.46 ± 2.67 [−6.26, −0.65]	−1.63 ± 1.27 [−2.4, −0.86]	−1.27 ± 2.07 [−2.09, −0.45]	−0.67 ± 1.38 [−1.16, −0.18]	−0.58 ± 1.86 [−1.19, 0.02]	−0.75 ± 1.81 [−1.34, −0.16]	−0.69 ± 1.29 [−1.12, −0.27]	0.01 ± 2.02 [−0.53, 0.55]	R^2^_c_= 0.13 ICC = 0.00	(a) F_[7|1361] = 20.9_	*p* < 0.001
	**3**	-	-	-	-	−3.12 ± 2.17 [−4.93, −1.31]	−2.53 ± 2.57 [−3.28, −1.77]	−1.63 ± 2.17 [−2.1, −1.16]	−0.76 ± 2.01 [−1.13, −0.39]	−0.79 ± 1.67 [−1.11, −0.48]	−0.32 ± 1.45 [−0.6, −0.03]	−0.13 ± 1.78 [−0.44, 0.17]	−0.11 ± 1.88 [−0.38, 0.16]		(b) F_[2|1361] = 0.5_	*p* = 0.584
	**4**	-	-	-	-	−3.79 ± 2.72 [−5.74, −1.85]	−3.82 ± 2.85 [−5.73, −1.9]	−1.26 ± 2.71 # [−2.11, −0.41]	−0.89 ± 2.80 [−1.67, −0.11]	0.04 ± 2.12 [−0.6, 0.68]	−0.03 ± 2.30 [−0.71, 0.65]	−0.40 ± 2.22 [−1.07, 0.26]	−0.07 ± 2.26 [−0.55, 0.41]		(c) F_[14|1361] = 1.5_	*p* = 0.115

Linear mixed model analysis. (a) Main effect: age category. (b) Main effect: performance level. (c) Interaction effect: age category x performance level. Bonferroni post hoc comparison: # significant difference compared to previous age category; * significant difference compared to performance level 2.

## Data Availability

All data are publicly available on Swimrankings.net.

## Abbreviations

The following abbreviations are used in this manuscript:

LMM	Linear mixed model
REML	Restricted maximum likelihood
ICC	Intra-class correlation coefficient
M	Males
F	Females
